# Combining Augmented Reality and 3D Printing to Improve Surgical Workflows in Orthopedic Oncology: Smartphone Application and Clinical Evaluation

**DOI:** 10.3390/s21041370

**Published:** 2021-02-15

**Authors:** Rafael Moreta-Martinez, Alicia Pose-Díez-de-la-Lastra, José Antonio Calvo-Haro, Lydia Mediavilla-Santos, Rubén Pérez-Mañanes, Javier Pascau

**Affiliations:** 1 Departamento de Bioingeniería e Ingeniería Aeroespacial, Universidad Carlos III de Madrid, 28911 Leganés, Spain; rmoreta@pa.uc3m.es (R.M.-M.); apose@ing.uc3m.es (A.P.-D.-d.-l.-L.); 2 Instituto de Investigación Sanitaria Gregorio Marañón, 28007 Madrid, Spain; calvoharo@yahoo.es (J.A.C.-H.); lydia.mediavilla@salud.madrid.org (L.M.-S.); rubenperez.phd@gmail.com (R.P.-M.); 3 Servicio de Cirugía Ortopédica y Traumatología, Hospital General Universitario Gregorio Marañón, 28007 Madrid, Spain; 4 Departamento de Cirugía, Facultad de Medicina, Universidad Complutense de Madrid, 28040 Madrid, Spain

**Keywords:** augmented reality, orthopedic oncology, 3D printing, computer-aided interventions, smartphone

## Abstract

During the last decade, orthopedic oncology has experienced the benefits of computerized medical imaging to reduce human dependency, improving accuracy and clinical outcomes. However, traditional surgical navigation systems do not always adapt properly to this kind of interventions. Augmented reality (AR) and three-dimensional (3D) printing are technologies lately introduced in the surgical environment with promising results. Here we present an innovative solution combining 3D printing and AR in orthopedic oncological surgery. A new surgical workflow is proposed, including 3D printed models and a novel AR-based smartphone application (app). This app can display the patient’s anatomy and the tumor’s location. A 3D-printed reference marker, designed to fit in a unique position of the affected bone tissue, enables automatic registration. The system has been evaluated in terms of visualization accuracy and usability during the whole surgical workflow. Experiments on six realistic phantoms provided a visualization error below 3 mm. The AR system was tested in two clinical cases during surgical planning, patient communication, and surgical intervention. These results and the positive feedback obtained from surgeons and patients suggest that the combination of AR and 3D printing can improve efficacy, accuracy, and patients’ experience.

## 1. Introduction

Orthopedic oncology involves the treatment of patients diagnosed with tumors in bone and soft tissues, including bone metastases, sarcomas, benign and cancerous tumors [1]. Even though these tumor types are uncommon and represent less than 1% of all new cancer diagnoses [2], they are considered a real challenge to clinicians as five-year survival rate is 50% [3].

The standard treatment of these tumors includes their complete surgical resection, ensuring a safety margin of healthy tissue, usually followed by external radiation therapy [4,5]. However, the local recurrence rate is up to 27% after a marginal resection [6]. For this reason, it is essential to efficiently plan the surgical approach preoperatively to improve surgical outcome, leave enough surgical margin, and reduce the risk of local recurrence or metastasis [7,8,9]. Preoperative imaging techniques, such as computed tomography (CT) or magnetic resonance imaging, allow estimating the size and location of the tumor and other surrounding anatomical structures [10,11]. However, this information is not always available during the intervention to better identify healthy and tumorous tissue differences. This means that the surgical procedure still depends on previous experience and subjective judgment of the surgeon to achieve complete tumor removal [12].

During the last decade, surgical navigation techniques have improved tumor resection accuracy, decreasing local occurrence, and improving surgical outcomes [13,14]. However, navigation systems present several limitations. They require point-based patient-to-image registration with anatomical landmarks that are difficult to identify during surgery. Besides, real-time navigation information is displayed on external screens, requiring the surgeon to move his attention away from the patient. 

Recent technologies, such as three-dimensional (3D) printing and augmented reality (AR), have increased their adoption in many medical areas with exciting benefits. 3D printing allows the rapid manufacturing of 3D solid objects from a digital file [15]. In the medical field, this includes patient-specific anatomical 3D biomodels useful for surgical planning and patient communication [16,17,18]. On the other hand, AR superimposes 3D virtual models onto physical objects in the real space, enabling the simultaneous interaction with both of them [19]. Physicians have found significant advantages when AR is applied to medical training [20], surgical navigation [21,22], or needle insertion [23]. These two technologies could overcome the limitations identified for surgical navigation by improving surgeon’s spatial perception of the anatomy and displaying relevant patient information on-site during surgical procedures.

Despite the number of medical specialties in which 3D printing and AR have been applied, there are not many publications reporting the use of these technologies in orthopedic oncology. Some studies have presented patient-specific 3D printed models of the affected bone and tumor for preoperative planning, reporting improved surgical outcomes in blood loss, operative time, and surgical incision [24,25]. Others propose an “in-house” workflow with desktop 3D printers designing patient-specific surgical guides to delimit the tumor or the osteotomy cutting plane during the surgical intervention [26,27]. Regardless of the clear benefit of these technologies, the 3D printing time and material required for large anatomical models limit their application [24]. On the other hand, AR-based navigation systems have been beneficial for improving the tumor’s location before and during surgery, since it can be overlaid on top of the patient’s anatomy when the target is difficult to identify [28,29]. However, one limitation of AR for surgical guidance is the registration of virtual and real data. Manual alignment [30] or electromagnetic tracking systems [31] have been tested to overcome this problem, although they provide limited accuracy and may involve extra instrumentation or time [32].

The combination of 3DP and AR could overcome these limitations and improve surgical outcomes. Previous studies have shown some initial results of this approach: Witowski et al. designed a workflow that reduced anesthetic time, morbidity and postoperative complications [33]; our group implemented an AR system that enabled automatic registration with a 3D-printed patient-specific surgical guide [34]. The integration of 3D printing and AR could be useful not only during the surgical intervention, but also in all surgical workflow stages [35]. In this study, we propose a new surgical framework including both technologies through the treatment process in orthopedics oncology. First, to support surgical planning, displaying the anatomical structures in three dimensions and real size. Then, assisting during patient communication to explain the pathology and treatment approach. Finally, providing surgical guidance by projecting the tumor and other structures over the patient’s anatomy. This solution has been developed as a AR-based smartphone application. It displays the patient’s anatomy and the tumor’s location using a 3D-printed reference marker designed to fit in a unique position of the affected bone tissue, thus enabling automatic registration. To evaluate the contribution of both technologies, we tested the proposed system on six 3D-printed patient-specific phantoms obtained from orthopedics tumors in a variety of anatomical locations. The solution was clinically evaluated during the whole surgical workflow in two patients, reporting physicians’ and patients’ perspectives using surveys.

## 2. Materials and Methods

**Figure 1:**
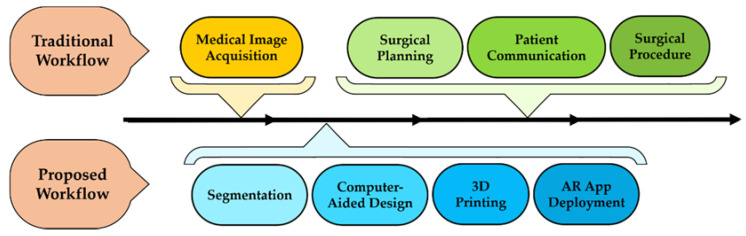
Prosed step by step orthopedics oncology medical workflow.

We describe the proposed orthopedics oncology surgical workflow in the following subsections. First, we present the patients involved in this study (Section 2.1). Then, Section 2.2. describes the preoperative image acquisition protocol and the design and manufacturing of the different tools. Next, we explain the proposed augmented reality system (Section 2.3) and the evaluation of its performance on patient-specific 3D-printed phantoms (Section 2.4). The last section shows the deployment of this technology at each stage of the surgical workflow (Section 2.5). A summary of the proposed surgical framework is presented in Figure 1 in comparison with the traditional one.

### 2.1. Patient Selection

We evaluated our proposal on data from six patients with tumors in bone or soft tissue treated by the Department of Orthopedic Surgery and Traumatology at Hospital General Universitario Gregorio Marañón. In order to maintain the anonymity of the patients, an alphanumeric code was assigned to each of them. The selected cases included tumors on different bones and anatomical regions (thorax, femur, hip and tibia) to ensure the added value of our workflow in a wide range of orthopedic oncology procedures. Table 1 summarizes the diagnosis, tumor size, and location for each patient. The study was performed in accordance with the principles of the 1975 Declaration of Helsinki as revised in 2013 and was approved by the Research Ethics Committee at Hospital General Universitario Gregorio Marañón. The anonymized patient data and pictures included in this paper are used after written informed consent was obtained from the participant and/or their legal representative, in which they approved the use of this data for dissemination activities, including scientific publications. 

### 2.2. Image Processing and Model Manufacturing

The proposed workflow includes steps such as medical image segmentation, computer-aided design, and 3D printing. From our experience, for each new patient, these three steps can be completed in less than 24 h.

#### 2.2.1. Medical Image Acquisition and Segmentation

A CT scan was acquired for each patient and used as reference image for segmentation. CT pixel size and time span between image acquisition and surgery are displayed in Table 2. We segmented the tumor and the surrounding bone tissue on 3D Slicer version 4.10 [36]. An initial bone mask was obtained with intensity thresholding and further refined with the islands tool, removing components such as the clinical bed. The tumor volume mask was extracted with manual painting and erasing tools. Finally, the segmentation masks were post-processed with hole filling (kernel size 7 × 7 × 3) to optimize 3D printing quality and minimize manufacturing time.

#### 2.2.2. Computer-Aided Design

The segmentation results were exported from 3D Slicer as virtual 3D models (stereo lithography files, STL) to be processed on Meshmixer software (Autodesk Inc., San Rafael, CA, USA). We used this program to design and extract several models for each patient: surgical guides for automatic registration, small bone fragments for surgical planning, and virtual models to display on the AR system.

Surgical guides are patient-specific tools designed to fit only in one specific location of the anatomy, usually bone tissue, during surgery. They can serve as physical models that mark the tumor limits during the surgery, or as cutting guides to resect the bone following the planes decided during surgical planning [26,37]. To design a surgical guide, the bone area on which it was intended to fit was selected, extracted, and extruded to create the surgical guide surface as a negative of the bone surface. In such a way, it had the specific curvature of the bone, perfectly fitting in that region and ensuring its unique positioning. The surgeons considered several parameters of the intervention to define the location of the surgical guide: the expected position and orientation of the patient, the line of sight of the physicians (that has to be preserved), and the surrounding tissue. Each guide included holes (Ø 5 mm) to attach it to the bone using screws, and a holder for the AR tracking marker that will allow automatic patient-to-image registration. The registration transformation was obtained with a previously developed 3D Slicer module [35].

**Figure 2:**
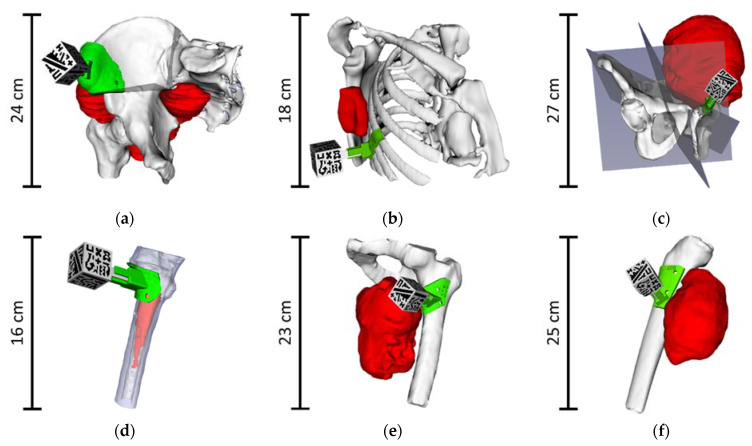
Virtual 3D models from patients: (**a**) AR3DP0002; (**b**) AR3DP0003; (**c**) AR3DP0004; (**d**) AR3DP0005, with some transparency in the bone to display the inner tumor; (**e**) AR3DP0006; (**f**) AR3DP0007. Tumors are represented in red, bones in white and surgical guides in green. Surgical cutting planes are illustrated in semi-transparent gray in the cases that required them: (**a**) and (**c**). The 3D-printed marker reference is positioned in the surgical guide.

For each case, we also extracted several bone fragments, smaller than the segmented bone structure, corresponding to the area of the bone in which the surgical guide was intended to fit. They were used to practice with the positioning of the surgical guide before and during the intervention. Finally, other models, such as cutting planes, were designed when required by the surgeons. Figure 2 shows the complete 3D models of the six patients included in this study with bone (white), tumor (red) and the surgical guide (green) in its corresponding position. When cutting planes were defined during surgical planning, they are displayed (semi-transparent gray). 

#### 2.2.3. 3D Printing

Once the 3D models had been defined, surgical guides and bone fragments were manufactured with desktop 3D printers. These tools were printed using a different technique depending on the expected use of the models. 

Surgical guides were fabricated using the stereolithography 3D printer Form 2 (Formlabs Inc., Somerville, MA, USA) with BioMed Clear V1 resin material (Formlabs Inc.). This resin is a USP class IV certified material, allowing contact inside the patient for long periods of time (more than a week) [38]. The 3D-printed surgical guides were sterilized before surgery with ethylene oxide (EtO) at 55° C [39]. The pieces will not deform under these sterilization conditions, since this material withstands high temperatures without distortion [40].

Fused deposition modeling (FDM) desktop 3D printers Ultimaker 3 Extended and Ultimaker S5 (Ultimaker B.V., Utrecht, The Netherlands) were used to manufacture all the other tools in polylactic acid (PLA). Bone fragments and a copy of the surgical guide were 3D printed using different color materials. A copy of each bone fragment was sterilized using EtO at low temperature (37 °C) (this sterilization has shown to avoid low deformation in PLA [41]) to be used as guidance during the surgical intervention. These tools will not be in contact with the patient. 

### 2.3. Augmented Reality System

**Figure 3:**
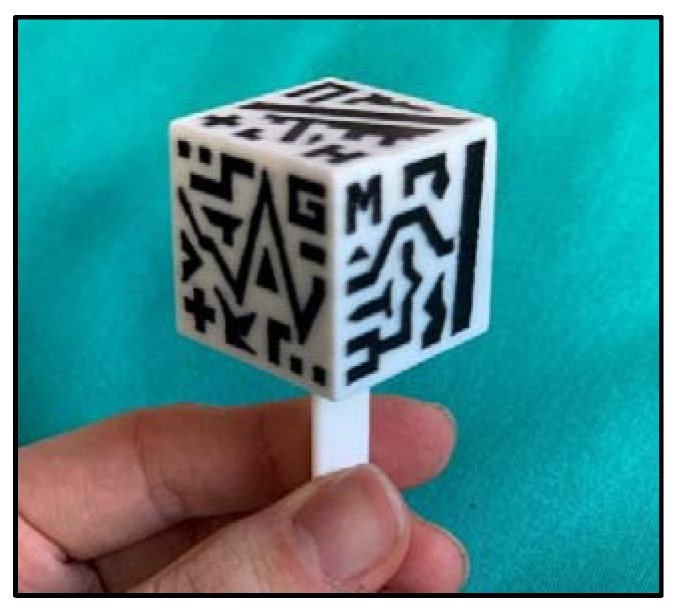
3D-printed augmented reality cubic marker used in the augmented reality system.

The smartphone application, *ARHealth*, was developed in Unity (version 2019.3), using C# programming language, and is compatible with Android and iOS devices. The app uses Vuforia SDK (Parametric Technology Corporation Inc., Boston, MA, USA) to identify the patterns of a cubic reference marker [35] in the smartphone camera field of view (FOV) and project the virtual models overlaid onto the real-world image. These models will be correctly registered with the patient since their relative coordinates are computed and stored using a previously developed 3D Slicer module [35], and then recovered by *ARHealth*. We designed the cubic marker (30 × 30 × 30 mm^3^) to contain unique patterns in black and white on each face (Figure 3). It also included an adaptor on one face to attach it to the corresponding holder in the surgical guide. This cubic reference was 3D printed in PLA using the dual extruder 3D Printer Ultimaker 3 Extended (Ultimaker B.V.) in white and black color materials, and it was sterilized with EtO at low temperature (37 °C) [41] before surgery. 

The smartphone application presents a main menu where the patient of interest is selected. Once chosen, it displays a second menu that offers three visualization modes: Demo, Clinic and Surgery.

**Figure 4:**
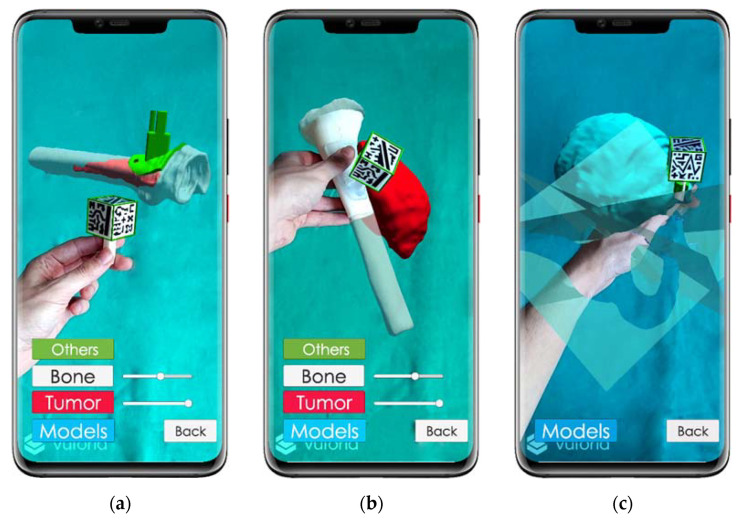
*ARHealth* smartphone application. (**a**) *Demo* mode using patient AR3DP0005 3D models, tumor is represented in red inside the bone, which is displayed in white with transparency texture; (**b**) Virtual visualization (tumor in red, bone in white with transparency texture) overlaid on top of the 3D-printed bone fragment (solid white) of patient AR3DP0007 using *Clinic* mode; (**c**) *Surgical* mode visualization of patient AR3DP0004 (tumor is represented in blue and cutting planes in semi-transparent green). A green frame surrounding the AR marker indicates that the reference is being tracked by the system.

- In the *Demo* mode, all the virtual 3D models are displayed around the AR marker, without any patient registration (Figure 4a). The AR marker can be rotated to show the virtual models from any point of view.
- *Clinic* mode displays the virtual 3D models in their corresponding position with respect to the surgical guide, which is registered to the cubic marker (Figure 4b). This mode is designed to be used with the surgical guide fixed on a 3D printed bone (or fragment), allowing for surgical planning and training.
- *Surgery* mode will be used during the actual surgical intervention. The surgical guide will be attached to the patient’s bone, solving the registration between the patient and the AR system. The main difference with *Clinic* mode is that those models that will be essential to the surgeon, such as tumor or cutting planes, are augmented on top of the patient to guide the operation in real-time. Besides, an occlusion texture could be assigned to the bone model within the app, covering the models behind it, providing the same visualization as if the actual bone was occluding these elements (Figure 4c).

The incorporation of a new case to the app is a simple process: once the necessary biomodels have been created, they are uploaded to the Unity project. The user interface and biomodel visualization parameters are then automatically updated. The smartphone app is then compiled and copied to our institutional smartphone. This procedure preserves data security since all patient related information is compiled in the app and cannot be exported or accessed. Besides, the app currently runs on our local smartphone devices.

### 2.4. Augmented Reality System Performance

**Figure 5:**
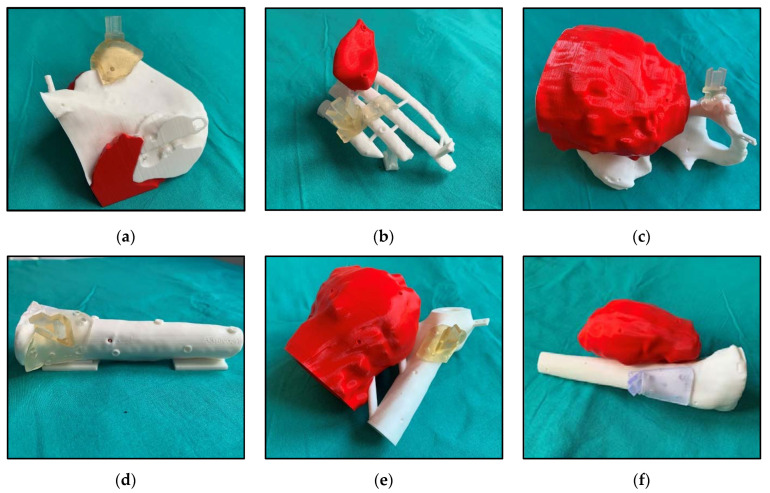
3D printed patient-specific phantoms from patient: (**a**) AR3DP0002; (**b**) AR3DP0003; (**c**) AR3DP0004; (**d**) AR3DP0005; (**e**) AR3DP0006; (**f**) AR3DP0007. Bones are in white, the tumors are in red, and the resin surgical guides are fitted on their corresponding position.

The performance of the proposed augmented reality system was evaluated on six 3D printed patient-specific phantoms, corresponding to each of the patients participating in the study. They were designed by selecting a representative region of the patient’s anatomy (including part of the bone and the tumor) and attaching to them some supports and bases. The supports joined parts that were not connected in the original anatomy in order to obtain a rigid phantom. The resulting models could stand over their base to maintain stability during the validation process. Eight small conical holes (Ø 4 mm × 3 mm depth) were added to the model surface for point-based registration and error measurement. Additionally, the surgical guides (that included the support for the AR marker) were also modified, adding three to five conical holes (Ø 4 mm × 3 mm depth), depending on the guide size. Those holes were used for error measurement. The phantoms dimensions are summarized in Table 3. These phantoms were 3D printed in PLA with the dual extruder FDM 3D printers in two different colors. The surgical guide specifically designed for validation was 3D printed in resin material to simulate the surgical intervention. Figure 5 displays the six resulting phantoms.

Surgical Guide Placement Error and the Augmented Reality Tracking Error were evaluated to assess the precision and accuracy of the AR system. A Polaris Spectra (Northern Digital Inc., Waterloo, ON, Canada) optical tracking system (OTS) managed by 3D Slicer was implemented as a gold-standard for the performance evaluation, following the methodology from a previous study [34]. Additionally, the distance range for marker detection of the system was also studied.

#### 2.4.1. Surgical Guide Positioning Error

This error was analyzed to assess the uniqueness of the surgical guide positioning on the bone. We attached the surgical guide to each phantom and recorded the position of the conical holes (from 3 to 5, depending on the guide) with a pointer tracked by the OTS. The Euclidean distance between the recorded coordinates and those obtained from the virtual models allowed us to determine the Surgical Guide Placement Error. This process was repeated five times by two users, removing and placing back again the guide on each phantom. We calculated the required point-based registration [42] between the 3D printed phantom and its virtual model using conical holes included in the validation phantom.

#### 2.4.2. Augmented Reality Tracking Error

**Figure 6:**
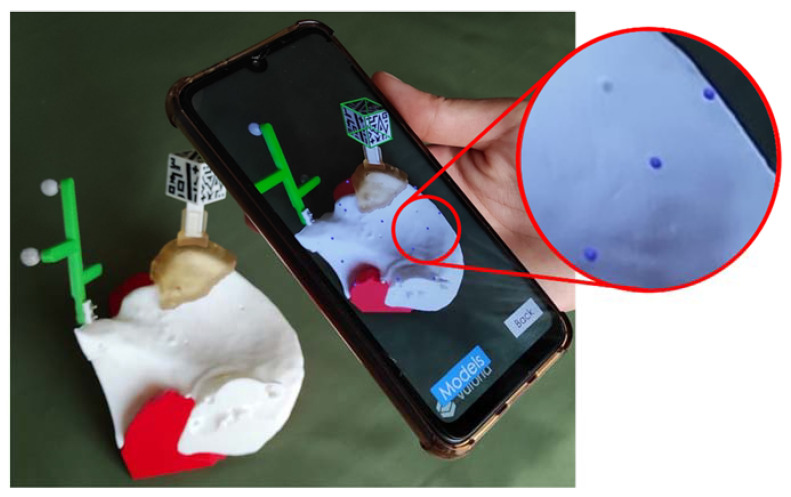
Phantom of patient AR3DP0002 (buttock tumor) and smartphone with the *ARHealth* validation app. The Augmented Reality Tracking Error validation spheres are augmented on the phantom surface in deep blue.

The overall AR system performance was determined using a modified version of the *ARHealth* app on a Google Pixel 4 XL smartphone (Alphabet Inc., Mountain View, CA, USA). First, the surgical guide was positioned and fixed on the phantom. Then, the AR reference marker was placed on the surgical guide enabling automatic registration between the AR system and the phantom. Once the AR system tracked the marker, 14 virtual spheres (Ø 3 mm) were randomly augmented on the surface of the 3D-printed phantom. Each user positioned the tip of a tracked pointer on the virtual spheres by looking at the smartphone screen, and that location was recorded with the OTS. The Euclidean distance between the recorded positions and true positions of the spheres was calculated to evaluate the Augmented Reality Tracking Error. Each experiment was repeated five times by two different users, removing and placing back the surgical guide. Figure 6 shows the phantom of the Patient AR3DP0002 (buttock tumor) and the smartphone with the modified version of *ARHealth* displaying the augmented spheres in deep blue.

### 2.5. Integration of the Augmented Reality System in the Surgical Workflow

The proposed AR System was implemented during the whole surgical workflow of patients AR3DP0006 and AR3DP0007. *ARHealth* was used at the three steps of the workflow: by the surgeons during surgical planning, to show the virtual anatomical models to the patients before surgery, and during the surgical intervention to display the tumor margins. Finally, a survey was designed to qualitatively record the opinion of the surgeons and the patients about the proposed AR-based system

During surgical planning, surgeons used *Demo* and *Clinic* modes from the *ARHealth* app installed in a Google Pixel 4 XL smartphone. First, the user selected *Demo* mode from the main menu, and holding the phone with one hand, he pointed with the camera to the AR marker to detect it. The virtual models, such as bone and tumor, were projected on the smartphone display. The AR app would track the cubic reference movements, and the virtual models moved according to the face detected. *Demo* mode was used to take a first glimpse of the case without needing any 3D printed biomodel. Then, *Clinic* mode was selected from the main menu of the app. This time, the surgeon took the 3D-printed surgical guide of the corresponding patient and fixed it into the PLA bone fragment. Then, the cubic marker was placed on the surgical guide, and with the smartphone camera pointing at it, the system displayed all the models with respect to the bone fragment. This mode was used to practice with the visualization before the surgery and with the surgical guide placement. The *Clinic* mode could also help analyze and discuss alternative strategies for the surgical procedures, compare possible approaches or instruct inexperienced surgeons.

During the last medical appointment with the patient prior to the surgery, physicians used *ARHealth* as a reinforcement to explain the tumor location and the treatment they were going to receive. With the *Clinic* mode selected on the smartphone, patients pointed with the camera to the cubic marker, which was already attached to the surgical guide and the bone fragment. They were able to rotate the marker and tailor the transparency of the models to more easily comprehend the details of their condition.

Our proposed technology was also evaluated as guidance for tumor resection. The system provided the tumor’s location thanks to the automatic registration between the reference marker and the surgical guide. An iPhone 6 (Apple Inc., Cupertino, CA, USA) was used as the AR-device during the procedure. The surgical intervention was developed without modifications until the tumor was completely removed, leaving the bone tissue exposed. After that, the smartphone was introduced in a sterilized case (CleanCase, Steridev Inc., Lansing, MI, USA) held by one surgeon. One of the surgeons opened the *ARHealth* app on the smartphone and selected the *Clinic* mode. The physician placed the surgical guide with the cubic marker attached on the sterile bone fragment and verified its position, ensuring that the app was working as expected. The next step was to place the surgical guide on the patient’s bone target area. Surgeons used the *Surgery* mode to visualize the bone and ensure the correct placement of the guide, validating the automatic registration. Then they fixed it in the bone with medical screws. One of the surgeons selected the occlusion bone mode and used the smartphone to project the tumor on the patient in real-time through the AR display. They could rotate the smartphone at any orientation to better evaluate the resection margin. Finally, they removed the surgical guide and continued with the procedure.

We designed two different questionnaire surveys, one for the patients and the other for the surgeons, to qualitatively assess the impact of the proposed workflow from their point of view and identify possible aspects that could be improved. Ten surgeons from the Department of Orthopedic Surgery and Traumatology filled the 16 questions from the surgeon’s questionnaire (Supplementary Materials Document S1). Survey’s questions were scored on a 5-point Likert scale. All participating surgeons were familiar with the AR system. The survey presented to the patients consisted of 6 questions (Supplementary Materials Document S2) and was completed by the two patients involved in the clinical validation.

## 3. Results

The detection of the AR cubic marker by the smartphone application was feasible and practically immediate if they were at an appropriate distance. We zoomed in and out the Google Pixel 4 XL and iPhone 6 smartphones, both under optimal and dim light conditions, to determine the distance at which the marker was detected (zoom in) and lost (zoom out). In all the cases, the AR marker was detected at 30 cm. Once the pattern was identified, it was possible to move the phone further away from the marker up to 50 cm and maintain the visualization (provided that the AR cube was always on the camera’s FOV).

### 3.1. Augmented Reality Performance

**Figure 7:**
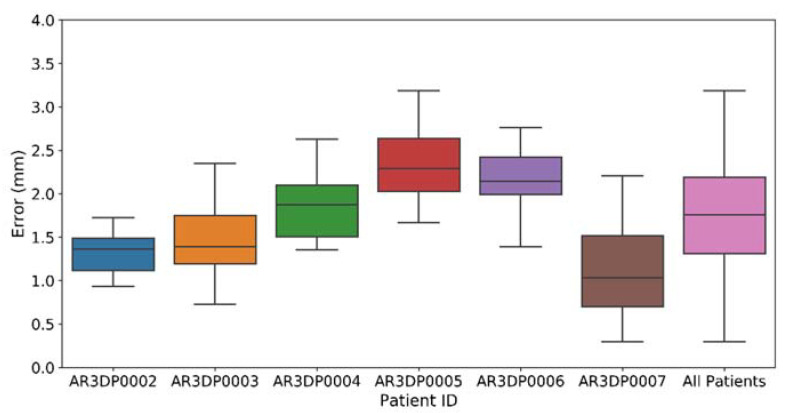
Surgical Guide Placement Error obtained for each patient phantom. The upper and lower limits for each box represent the first and third quartile of the dataset, and the middle line indicates the median. The whiskers stand for the highest and lowest values (±1.5 times the standard deviation).

Figure 7 displays the results obtained during the validation of the Surgical Guide Placement Error for each phantom by two users and five repetitions each. The last column includes the measures on all phantoms. The error obtained for Patient AR3DP0005 could not be analyzed due to technical errors during the corresponding evaluation experiment. The overall Surgical Guide Placement Error was 1.75 ± 0.61 mm. 

**Figure 8:**
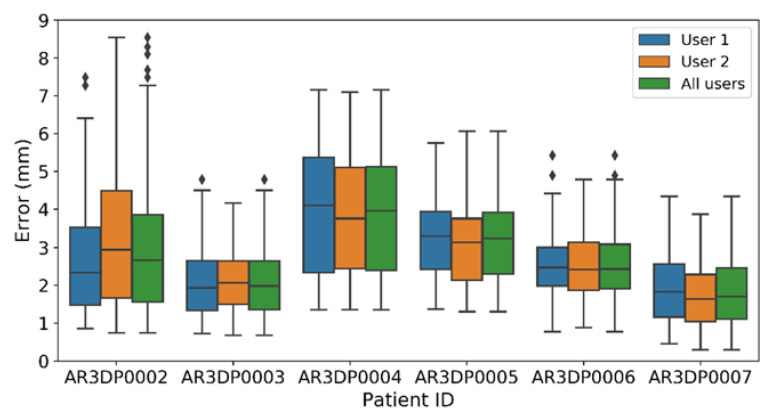
Augmented Reality Tracking Error for all the patients separated by user. The upper and lower limits for each box represent the first and third quartile of the dataset, the middle line indicates the median. The whiskers stand for the highest and lowest values (±1.5 times the standard deviation).

The Augmented Reality Tracking Error validation experiments were designed to demonstrate the system’s accuracy and independence from the user. Figure 8 represents the error values for five repetitions on each phantom. The minimum mean error is obtained for case AR3DP0007 (2.1 ± 0.9 mm) and the maximum for AR3DP0004 (4.2 ± 1.5 mm). To determine the user-dependency of the error values, we performed a paired t-test comparing the data obtained by both users in each phantom under the null hypothesis that there is not a significant difference between their results, obtaining a *p*-value > 0.05 in all cases. Overall, the mean error of the system was 2.80 ± 0.98 mm.

### 3.2. Integration of the Augmented Reality System in the Surgical Workflow

**Figure 9:**
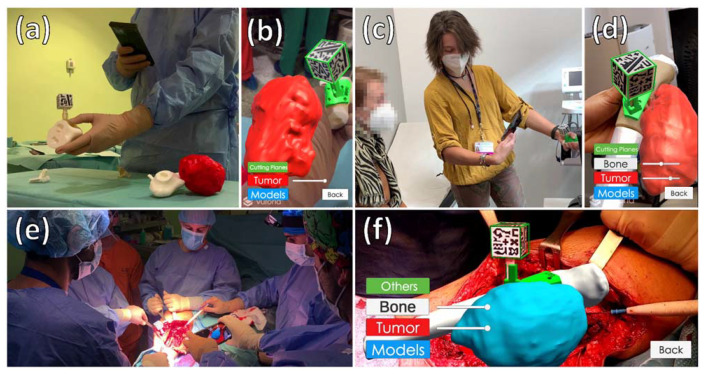
Integration of the augmented reality system at each step of the medical workflow. (**a**,**b**) Physician using *ARHealth* during surgical planning of patient AR3DP0006; (**c**,**d**) Medical staff explaining patient AR3DP0007 her condition using *ARHealth*; (**e**) One physicians using *ARHealth* during surgical intervention of patient AR3DP0007 after the surgical guide was placed on the patient, and other surgeon delimiting surgical margin while looking at the AR-display. (**b**,**d**,**f**) Smartphone visualization at the same moment of (**a**,**c**,**e**), respectively.

The integration of *ARHealth* was feasible on all the surgical workflow steps for patients AR3DP0006 and AR3DP007. During the preoperative planning, surgeons used *Demo* and *Clinic* mode from the *ARHealth* app to reinforce their knowledge about each patient case. It allowed physicians to discuss the surgical approach anywhere in the hospital, thanks to the portability of the system (Figure 9a,b). During the last clinical appointment, both patients held the smartphone and tracked the AR marker easily. Patients understood how this technology worked with just one explanation from the physician, and both used the system for approximately 5 min. Figure 9c,d shows one of the patients using the AR system during the clinical appointment. Detection of the cubic marker was fast, and no problems were encountered during these two steps of the medical workflow.

During the surgical intervention, the smartphone was placed on the sterile case without complications. The 3D-printed bone fragment was useful in both cases to verify the position of the surgical guide before placing it on the real bone. Moreover, surgical guides fitted as planned in the target area of the patient’s bone, allowing a successful registration between the AR system and the patient’s anatomy. The virtual models were projected on the patients in their expected location. Neither blood nor different light conditions interfered with the detection of the cubic marker, and *ARHealth* could display the tumor to better evaluate the surgical margins. Additionally, the 3D-printed reference stayed fixed on the surgical guide adaptor and was easily removed when it was no longer needed. The picture of Figure 9e,f was acquired during the surgical intervention of Patient AR3DP0007. One surgeon is holding the smartphone in the sterile case projecting the tumor virtual model on top of the patient after tumor resection to delimit tumor margin.

During AR3DP0006 surgical intervention, some main arteries interfered with the cubic marker detection after placing it into the surgical guide. However, thanks to the cubic shape of the tracking reference, the AR system detected other uncovered faces of the cubic marker, projecting the anatomical virtual models in the right position on the patient. 

Nine orthopedic surgeons answered the proposed survey. Five of them had prior experience with AR. Table 4 reveals the individual scores obtained from each user. The last row and column represent the average scores per question and surgeon, and the left-most column is a condensed form of the questions asked. The total average score obtained in the survey, considering the questions related to the smartphone application and the usability of the proposed workflow, was 4.5 out of 5. Regarding the medical fields that they considered could benefit the most from this technology as it is right now, they all selected oncologic surgery. 78% of them additionally answered orthopedic surgery and 67% chose neurosurgery, plastic surgery or minimally invasive surgery. The 44% of the physicians also picked cardiac surgery.

Patient’s survey was answered by the two patients for whom the whole medical workflow was deployed (Table 5). None of them had prior experience with AR or had even seen a 3D model of their body before. Both patients gave the maximum score to the *ARHealth* system and preferred AR in the explanation of their pathology rather than a 2D image or the standard surgeon’s description.

## 4. Discussion

In this study, we present and evaluate a novel framework deployed in orthopedic oncology combining AR and 3D printing technologies to assist surgeons during different stages of the surgical workflow. Our system supports surgical planning, enhances patient communication, and provides guidance during surgical interventions. A smartphone-based AR application has been developed to visualize the patient’s anatomy and the tumor locations using a 3D-printed reference marker. Automatic registration between virtual and real world is achieved by patient-specific surgical guides (with a support for the reference marker) that fit in a unique region of the affected bone tissue. The precision of the system has been analyzed using the clinical data from six patients, and the feasibility has been evaluated during the whole surgical workflow on two of them. 

3D printing still has significant limitations, such as long 3D printing time of large pieces and a lot of material waste in orthopedic oncology [24]. In this study, these problems have been tackled by 3D printing just small models, such as delimit fragments of the affected areas, and displaying the complete biomodels with AR. This method could be an alternative to reduce material cost and 3D printing time.

The main limitation of AR in the medical field is the required image registration between real and virtual data [43]. Some studies try to solve this issue with a manual registration [30], which might not be the best option in many cases. We adapted the automatic registration technique presented in [34], proposing a more general approach. The 3D-printed surgical guide has now a marker holder attached to it. This allows the attachment of an AR marker when the surgical guide is fixed on the patient without removing the whole surgical guide. Additionally, we have demonstrated that this guide is placed in the target position with a mean error below 1.80 mm in six patient-specific phantoms of different bone types and areas. These results are comparable with those reported in [34]. The low error indicated that this registration method could be reliable for AR systems applied to orthopedic oncology.

When evaluating the Augmented Reality Tracking Error on six patient-specific phantoms the overall error was 2.80 mm. These results are similar to those reported in [34], using Microsoft HoloLens, and in [44], using a tablet-based system. However, a relevant fact is that in [34] the AR marker was two-dimensional, limiting the mobility of the AR device. Our study obtains similar tracking error results, but with a 3D printed cubic marker that can be tracked from different points of view, providing freedom of movement, a crucial aspect specially in the OR. 

The AR performance achieved comes from an accumulation of different error sources, such as the accuracy of the 3D printers, the localization of the control points, the intrinsic error of the OTS and the registration error. However, the low error obtained in our system encourages us to believe that virtual models can be displayed with enough accuracy on top of the patient to improve different steps of the surgical workflow.

The Augmented Reality Tracking Error results reveal increased variability for the biggest phantoms, suggesting an increasing error at larger distances. Nonetheless, this is common to all navigation systems [45]. Phantom AR3DP0002 and Phantom AR3DP0004 were the largest in our experiments and, therefore, had more error evaluation positions further away from the origin (the AR marker). Consequently, the error results for those phantoms are higher both in average and standard deviation. Although existing, this correlation will not affect during this type of surgeries, on which the working volumes are limited around the surgical guide. Anyway, this factor must be considered in each case.

The proposed AR system was favorably tested during the complete medical workflow of two patients. The visualization of virtual 3D models of the patients was feasible using the smartphone and the tracking marker during surgical planning, patient communication and surgical intervention. The general opinion of the surgeons is that the system would be very useful to establish a preoperative plan more confidently (by examining the case in three dimensions before surgery). The solution is portable, not requiring a personal computer. The importance of patient communication in surgical interventions was already highlighted in [46]. In our case, both patients welcomed this technology to understand their situation better, and surgeons found it very useful to accompany their explanations of the pathology and surgical approach. 

Additionally, the proposed methodology was easily integrated during the two clinical interventions. The system displayed the corresponding tumor position on top of the patient with virtual anatomical elements, boosting surgeons’ confidence to verify that the tumor has been adequately resected. The physicians believed this visualization could be beneficial in tumor resection surgeries. Moreover, they suggested that this technology could also be advantageous to guide osteotomy cutting planes. AR visualization offers advantages compared to image guided information shown in standard displays, since the actual anatomy captured by the camera is combined with digital models in a natural way for the user. Our experience is still limited, so further evaluation is required in a larger sample. The system accuracy is good but may not be enough to replace surgical navigation techniques. 

Finally, the survey’s results revealed an overall great acceptance of this system in the hospital and endorse the applicability of our proposal from the clinical perspective, promoting further research on this area. The questions related to the medical fields that could potentially benefit from this system open the future applications spectrum of this technology.

One of our system’s limitations is that we created the 3D virtual biomodels based on an CT acquired several weeks before the surgery, and the tumors may have grown, reduced or moved in that time lapse [47]. Nevertheless, this is a limitation for any navigation system based on preoperative images. Another limitation is that, to use the *ARHealth* app, one hand must continuously hold the smartphone. A mechanical arm, holding the smartphone, could be incorporated into the OR to address this issue. Even the system could be running on a head-mounted display, such as Microsoft Hololens 2, to free the surgeon’s hands in the procedures and give him/her more maneuverability during surgical intervention.

With regard to the expansion of our system to other hospitals, some extra security protocols should be applied to the smartphone application in order to preserve patients’ privacy. This could be implemented with OAuth 2.0 protocol [48] as an authorization framework to limit the access to the app to only qualified personnel.

In conclusion, we have shown the benefits that the combination of AR and 3D printing can bring to orthopedic oncology surgery by evaluating the proposed AR system in patient-specific 3D-printed phantoms and at each stage of the surgical workflow. We believe that this work serves as a baseline for developing more AR and 3D printing systems for surgical guidance, training, and patient communication.

## Figures and Tables

**Table 1:** Patient demographics involved in this study.

Case ID	Gender/Age	Diagnosis	Tumor Location	Tumor Size [cm]
**AR3DP0002**	M/62	Myxofibrosarcoma	Right buttock	18 × 19 × 17
**AR3DP0003**	F/71	Liposarcoma	Right periscapular region	3 × 3 × 6
**AR3DP0004**	M/19	Ewing Sarcoma	Left iliac crest	13 × 19 × 16
**AR3DP0005**	F/66	Fibrous dysplasia	Left femur	4 × 2 × 8
**AR3DP0006**	M/79	Myxofibrosarcoma	Left thigh	10 × 15 × 12
**AR3DP0007**	F/84	Undifferentiated pleomorphic sarcoma	Right calf	10 × 8 × 14

**Table 2:** Resolution of the CT scan acquired for each patient and time span between image acquisition and surgery.

Case ID	CT Resolution [mm]	CT–Surgery Time Span [Days]
**AR3DP0002**	0.93 × 0.93 × 1.00	13
**AR3DP0003**	1.31 × 1.31 × 3.00	101
**AR3DP0004**	0.98 × 0.98 × 2.50	137
**AR3DP0005**	0.78 × 0.78 × 0.80	92
**AR3DP0006**	1.10 × 1.10 × 5.00	94
**AR3DP0007**	1.13 × 1.13 × 3.00	83

**Table 3:** Size dimensions of the manufactured patient-specific 3D-printed phantoms.

Case ID	Phantom Dimension [cm]
AR3DP0002	17 × 15 × 13
AR3DP0003	12 × 11 × 9
AR3DP0004	22 × 22 × 19
AR3DP0005	16 × 10 × 5
AR3DP0006	17 × 15 × 10
AR3DP0007	22 × 12 × 11

**Table 4:** Surgeons’ survey scores.

Questions.	Individual Scores (per surgeon)						Avg. Score
	1	2	3	4	5	6	
1. AR in surgeries (general)	5	4	5	5	3	5	4.5
2. AR in surgeon’s operations (general)	5	4	3	3	4	5	4.0
3. DEMO: surgeon understanding	5	4	5	5	4	5	4.7
4. DEMO: surgical planning	5	4	5	5	5	5	4.8
5. DEMO: patient communication	5	5	5	5	5	5	5.0
6. CLINIC: PLA bone fragment	5	4	4	5	4	5	4.5
7. CLINIC: practice with AR	5	3	4	5	5	5	4.5
8. CLINIC: patient communication	5	3	5	5	3	5	4.3
9. SURGERY: tumor location	5	5	4	5	4	5	4.7
10. SURGERY: increase of accuracy	5	4	4	5	5	5	4.7
11. SURGERY: phone case	5	4	5	5	5	5	4.8
12. GENERIC: easiness of interpretation	5	5	4	5	4	5	4.7
13. GENERIC: patient communication	5	5	5	5	5	5	5.0
14. GENERIC: surgeon’s confidently	4	4	5	5	4	5	4.5
15. GENERIC: use this workflow	5	4	4	5	4	5	4.5
Avg. Score	4.9	4.1	4.5	4.9	4.3	5	4.5

**Table 5:** Patients’ survey scores.

Questions	Individual Scores (per patient)		Avg. Score
	1	2	
1. Pathology understanding before *ARHealth*	2	4	3
2. Pathology understanding after *ARHealth*	5	5	5
3. General opinion about AR	5	5	5

## Data Availability

Data is contained within the article or supplementary material.

## Supplementary Materials

The following are available online at https://www.mdpi.com/1424-8220/21/4/1370/s1, Document S1: Questionnaire given to surgeons to validate the AR system, Document S2: Questionnaire given to patients involved in this study to validate the AR system.

## References

[1] Fletcher C.D., Unni K., Mertens F. (2002). Pathology and Genetics of Tumours of Soft Tissue and Bone.

[2] Hui J.Y.C. (2016). Epidemiology and Etiology of Sarcomas. Surg. Clin. N. Am..

[3] Clark M.A., Fisher C., Judson I., Thomas J.M. (2005). Soft-Tissue Sarcomas in Adults. N. Engl. J. Med..

[4] Casali P.G., Jost L., Sleijfer S., Verweij J., Blay J.-Y. (2008). Soft tissue sarcomas: ESMO clinical recommendations for diagnosis, treatment and follow-up. Ann. Oncol. Off. J. Eur. Soc. Med. Oncol..

[5] (2014). Bone sarcomas: ESMO Clinical Practice Guidelines for diagnosis, treatment and follow-up. Ann. Oncol. Off. J. Eur. Soc. Med. Oncol..

[6] Jeys L., Grimer R., Carter S., Tillman R., Abudu S. (2012). Outcomes of Primary Bone Tumours of the Pelvis—The Roh Experience. Orthop. Proc..

[7] Kawaguchi N., Ahmed A.R., Matsumoto S., Manabe J., Matsushita Y. (2004). The Concept of Curative Margin in Surgery for Bone and Soft Tissue Sarcoma. Clin. Orthop. Relat. Res..

[8] Gundle K.R., Kafchinski L., Gupta S., Griffin A.M., Dickson B.C., Chung P.W., Catton C.N., O’Sullivan B., Wunder J.S., Ferguson P.C. (2018). Analysis of Margin Classification Systems for Assessing the Risk of Local Recurrence After Soft Tissue Sarcoma Resection. J. Clin. Oncol..

[9] Qureshi Y.A., Huddy J.R., Miller J.D., Strauss D.C., Thomas J.M., Hayes A.J. (2012). Unplanned excision of soft tissue sarcoma results in increased rates of local recurrence despite full further oncological treatment. Ann. Surg. Oncol..

[10] Smolle M.A., Andreou D., Tunn P.-U., Szkandera J., Liegl-Atzwanger B., Leithner A. (2017). Diagnosis and treatment of soft-tissue sarcomas of the extremities and trunk. EFORT Open Rev..

[11] Atesok K., Galos D., Jazrawi L.M., Egol K.A. (2015). Preoperative Planning in Orthopaedic Surgery. Current Practice and Evolving Applications. Bull. Hosp. Jt. Dis..

[12] Cartiaux O., Docquier P.-L., Paul L., Francq B.G., Cornu O.H., Delloye C., Raucent B., Dehez B., Banse X. (2008). Surgical inaccuracy of tumor resection and reconstruction within the pelvis: An experimental study. Acta Orthop..

[13] Jeys L., Matharu G.S., Nandra R.S., Grimer R.J. (2013). Can computer navigation-assisted surgery reduce the risk of an intralesional margin and reduce the rate of local recurrence in patients with a tumour of the pelvis or sacrum?. Bone Jt. J..

[14] Cho H.S., Oh J.H., Han I., Kim H.-S. (2012). The outcomes of navigation-assisted bone tumour surgery. J. Bone Jt. Surg. Br..

[15] Ventola C.L. (2014). Medical Applications for 3D Printing: Current and Projected Uses. P T.

[16] Barber S.R., Wong K., Kanumuri V., Kiringoda R., Kempfle J., Remenschneider A.K., Kozin E.D., Lee D.J. (2018). Augmented Reality, Surgical Navigation, and 3D Printing for Transcanal Endoscopic Approach to the Petrous Apex. OTO Open Off. Open Access J. Am. Acad. Otolaryngol. Neck Surg. Found..

[17] Yoo S.-J., Thabit O., Kim E.K., Ide H., Yim D., Dragulescu A., Seed M., Grosse-Wortmann L., van Arsdell G. (2016). 3D printing in medicine of congenital heart diseases. 3D Print. Med..

[18] Colaco M., Igel D.A., Atala A. (2018). The potential of 3D printing in urological research and patient care. Nat. Rev. Urol..

[19] Shuhaiber J.H. (2004). Augmented Reality in Surgery. Arch. Surg..

[20] Khor W.S., Baker B., Amin K., Chan A., Patel K., Wong J. (2016). Augmented and virtual reality in surgery—the digital surgical environment: Applications, limitations and legal pitfalls. Ann. Transl. Med..

[21] Inoue D., Cho B., Mori M., Kikkawa Y., Amano T., Nakamizo A., Yoshimoto K., Mizoguchi M., Tomikawa M., Hong J. (2013). Preliminary Study on the Clinical Application of Augmented Reality Neuronavigation. J. Neurol. Surg. A. Cent. Eur. Neurosurg..

[22] García-Mato D., Moreta-Martinez R., García-Sevilla M., Ochandiano S., García-Leal R., Pérez-Mañanes R., Calvo-Haro J.A., Salmerón J.I., Pascau J. (2020). Augmented reality visualization for craniosynostosis surgery. Comput. Methods Biomech. Biomed. Eng. Imaging Vis..

[23] Heinrich F., Joeres F., Lawonn K., Hansen C. (2019). Comparison of Projective Augmented Reality Concepts to Support Medical Needle Insertion. IEEE Trans. Vis. Comput. Graph..

[24] Punyaratabandhu T., Liacouras P.C., Pairojboriboon S. (2018). Using 3D models in orthopedic oncology: Presenting personalized advantages in surgical planning and intraoperative outcomes. 3D Print. Med..

[25] Jiang M., Chen G., Coles-Black J., Chuen J., Hardidge A. (2020). Three-dimensional printing in orthopaedic preoperative planning improves intraoperative metrics: A systematic review. ANZ J. Surg..

[26] Arnal-Burró J., Pérez-Mañanes R., Gallo-del-Valle E., Igualada-Blazquez C., Cuervas-Mons M., Vaquero-Martín J. (2017). Three dimensional-printed patient-specific cutting guides for femoral varization osteotomy: Do it yourself. Knee.

[27] Sternheim A., Gortzak Y., Kolander Y., Dadia S., Dipaola M., Wodajo F.M.B.T. (2019). Chapter 15—3D Printing in Orthopedic Oncology.

[28] Souzaki R., Ieiri S., Uemura M., Ohuchida K., Tomikawa M., Kinoshita Y., Koga Y., Suminoe A., Kohashi K., Oda Y. (2013). An augmented reality navigation system for pediatric oncologic surgery based on preoperative CT and MRI images. J. Pediatr. Surg..

[29] Abdel Al S., Chaar M.K.A., Mustafa A., Al-Hussaini M., Barakat F., Asha W. (2020). Innovative Surgical Planning in Resecting Soft Tissue Sarcoma of the Foot Using Augmented Reality With a Smartphone. J. Foot Ankle Surg..

[30] Pratt P., Ives M., Lawton G., Simmons J., Radev N., Spyropoulou L., Amiras D. (2018). Through the HoloLens^TM^ looking glass: Augmented reality for extremity reconstruction surgery using 3D vascular models with perforating vessels. Eur. Radiol. Exp..

[31] Kuhlemann I., Kleemann M., Jauer P., Schweikard A., Ernst F. (2017). Towards X-ray free endovascular interventions—Using HoloLens for on-line holographic visualisation. Healthc. Technol. Lett..

[32] Tang R., Ma L., Xiang C., Wang X., Li A., Liao H., Dong J. (2017). Augmented reality navigation in open surgery for hilar cholangiocarcinoma resection with hemihepatectomy using video-based in situ three-dimensional anatomical modeling: A case report. Medicine.

[33] Witowski J., DaRocha S., Kownacki Ł, Pietrasik A., Pietura R., Banaszkiewicz M., Kamiński J., Biederman A., Torbicki A., Kurzyna M. (2019). Augmented reality and three-dimensional printing in percutaneous interventions on pulmonary arteries. Quant. Imaging Med. Surg..

[34] Moreta-Martinez R., García-Mato D., García-Sevilla M., Pérez-Mañanes R., Calvo-Haro J., Pascau J. (2018). Augmented reality in computer-assisted interventions based on patient-specific 3D printed reference. Healthc. Technol. Lett..

[35] Moreta-Martinez R., García-Mato D., García-Sevilla M., Pérez-Mañanes R., Calvo-Haro J.A., Pascau J. (2020). Combining Augmented Reality and 3D Printing to Display Patient Models on a Smartphone. JoVE.

[36] Pieper S., Halle M., Kikinis R. 3D Slicer. Proceedings of the 2004 2nd IEEE International Symposium on Biomedical Imaging: Nano to Macro (IEEE Cat No. 04EX821).

[37] Pérez-Mañanes R., Burró J.A., Manaute J.R., Rodriguez F.C., Martín J.V. (2016). 3D Surgical Printing Cutting Guides for Open-Wedge High Tibial Osteotomy: Do It Yourself. J. Knee Surg..

[38] (2012). The United States Pharmacopeia.

[39] Zislis T., Martin S.A., Cerbas E., Heath J.R., Mansfield J.L., Hollinger J.O. (1989). A scanning electron microscopic study of in vitro toxicity of ethylene-oxide-sterilized bone repair materials. J. Oral Implantol..

[40] Sharma N., Cao S., Msallem B., Kunz C., Brantner P., Honigmann P., Thieringer F.M. (2020). Effects of Steam Sterilization on 3D Printed Biocompatible Resin Materials for Surgical Guides-An Accuracy Assessment Study. J. Clin. Med..

[41] Moreta-Martinez R., Calvo-Haro J.A., Pérez-Mañanes R., García-Sevilla M., Mediavilla-Santos L., Pascau J. (2020). Desktop 3D Printing: Key for Surgical Navigation in Acral Tumors?. Appl. Sci..

[42] Arun K.S., Huang T.S., Blostein S.D. (1987). Least-Squares Fitting of Two 3-D Point Sets. IEEE Trans. Pattern Anal. Mach. Intell..

[43] Wayne M., Ryan J., Stephen S. (2019). Virtual and Augmented Reality Applications in Medicine and Surgery The Fantastic Voyage is here. Anat. Physiol. Curr. Res..

[44] Wen R., Chng C.B., Chui C.K. (2017). Augmented reality guidance with multimodality imaging data and depth-perceived interaction for robot-assisted surgery. Robotics.

[45] Fitzpatrick J., West J., Maurer C. (1998). Predicting error in rigid-body point-based registration. IEEE Trans. Med Imaging.

[46] Street R.L.J., Makoul G., Arora N.K., Epstein R.M. (2009). How does communication heal? Pathways linking clinician-patient communication to health outcomes. Patient Educ. Couns..

[47] Kaiser D., Hoch A., Kriechling P., Graf D.N., Waibel F.W.A., Gerber C., Müller D.A. (2020). The influence of different patient positions on the preoperative 3D planning for surgical resection of soft tissue sarcoma in the lower limb-a cadaver pilot study. Surg. Oncol..

[48] Fett D., Küsters R., Schmitz G. (2016). A Comprehensive Formal Security Analysis of OAuth 2.0. Proceedings of the 2016 ACM SIGSAC Conference on Computer and Communications Security.

