# Supplementary material for: Combining Augmented Reality and 3D Printing to Improve Surgical Workflows in Orthopedic Oncology: Smartphone Application and Clinical Evaluation
Source: Sensors (Basel). 2021 Feb 15;21(4):1370. doi: 10.3390/s21041370 (PMC7919470; doi:10.3390/s21041370)
Supplement: Supplementary file 1 [file sensors-21-01370-s001.zip › Supplementary Material/DocumentS1_SurgeonsSurvey.pdf]

Survey to surgeons for ARHealth project

Name	
Date	
Medical specialty	
Had you ever used AR before?	☐ Yes ☐ No

Question	Rating
1. What do you think about the use of AR during surgeries?	1 2 3 4 5
2. Would you like to use AR in your surgeries?	1 2 3 4 5
**DEMO MODE**	
3. Do you think that the 3D visualization obtained in the DEMO mode could help the surgeon to better understand the pathology?	1 2 3 4 5
4. Do you think that the DEMO mode could facilitate the surgical planning?	1 2 3 4 5
5. Do you think that the DEMO mode could be useful to assist during patient communication?	1 2 3 4 5
**CLINIC MODE**	
6. Do you think that 3D printing a bone fragment for the app CLINIC mode could be useful for practicing surgical guide placement before surgical interventions?	1 2 3 4 5
7. Do you think that the app CLINIC mode would be useful to get used to AR visualization before surgeries?	1 2 3 4 5
8. Do you think that the app CLINIC mode would be useful to explain the patient his/her pathology?	1 2 3 4 5
**SURGERY MODE**	
9. Do you think that the SURGERY mode would facilitate tumor location in relation to the visible structures of the patient's body during operations?	1 2 3 4 5
10. Do you think that ARHealth could increase the accuracy of orthopedic oncological surgeries?	1 2 3 4 5
11. What do you think about the AR visualization on a smartphone/tablet in sterilizable case suitable for its use in the OR?	1 2 3 4 5
**GENERIC QUESTIONS**	
12. Do you think that the AR information provided through the virtual models projected over the image, is easy to interpret?	1 2 3 4 5
13. Do you think this technology could improve patient communication?	1 2 3 4 5
14. Do you think that AR could enhance the surgeons' confidently during surgical interventions?	1 2 3 4 5
15. Would you include this technology in your workflow?	1 2 3 4 5
16. Which medical areas do you think that could benefit the most from this technology?	☐ Orthopedic surgery ☐ Cardiac surgery<br>☐ Oncologic surgery ☐ Neurosurgery<br>☐ Minimally Invasive Surgery (MIS) ☐ Plastic surgery<br>☐ Others:

Observations	
