# Supplementary material for: Combining Augmented Reality and 3D Printing to Improve Surgical Workflows in Orthopedic Oncology: Smartphone Application and Clinical Evaluation
Source: Sensors (Basel). 2021 Feb 15;21(4):1370. doi: 10.3390/s21041370 (PMC7919470; doi:10.3390/s21041370)
Supplement: Supplementary file 1 [file sensors-21-01370-s001.zip › Supplementary Material/DocumentS2_PatientsSurvey.pdf]

Survey to patients for ARHealth project

Patient ID		
Date		
Had you ever seen a 3D model of any part of your body before?	☐ Yes	☐ No

Question	Rating				
1. How easy was to understand your situation from the medical images and the surgeon explanation?	1	2	3	4	5
2. How easy was to understand your situation with the AR smartphone app?	1	2	3	4	5
3. What is your general opinion about AR?	1	2	3	4	5
4. Do you prefer the explanation of your pathology with AR or as traditionally?	☐ AR<br>☐ Traditional explanation				

**Observations**	
